# Implementation of a National Synthetic Data Infrastructure for Privacy-Preserving Analytical Workflows in the Czech National Health Information System: Development and Evaluation Study

**DOI:** 10.2196/80683

**Published:** 2026-11-10

**Authors:** Martin Komenda, Daniel Klimeš, Ales Tichopad, Jiří Jarkovský, Ondřej Májek, Jan Mužík, Miloš Táborský, Vladimíra Těšitelová, Jan Habětínek, Ladislav Dušek

**Affiliations:** 1 Institute of Health Information and Statistics of the Czech Republic, Palackého náměstí 4, P.O. BOX 60, Praha, 128 00, Czech Republic, 420 725174940; 2 Institute of Biostatistics and Analyses, Faculty of Medicine, Masaryk University, Brno, Czech Republic; 3 Faculty of Biomedical Engineering, Czech Technical University in Prague, Prague, Czech Republic; 4 Czech Society of Cardiology, Prague, Czech Republic; 5 Faculty of Health Studies, Jan Evangelista Purkyně University in Ústí nad Labem, Ústí nad Labem, Czech Republic; 6 AstraZeneca, Czech Republic, Prague, Czech Republic

**Keywords:** synthetic data, health data sharing, European Health Data Space, General Data Protection Regulation, National Health Information System

## Abstract

**Background:**

Demand for health data to support research, innovation, and evidence-based policymaking is increasing, but access to patient-level data is constrained by privacy and data protection requirements. To address this challenge, the Czech Republic developed a national synthetic data infrastructure within the National Health Information System (NHIS). The infrastructure aims to support privacy-preserving secondary use of health data by enabling the development and validation of analytical workflows without providing direct access to operational patient-level databases.

**Objective:**

This study describes the design, implementation, governance framework, and operational evaluation of the Czech NHIS synthetic data infrastructure as a national platform for privacy-preserving secondary use of health data and for the development of reproducible analytical workflows. The system strongly supports the continuing relevance and importance of national infrastructures during the transition to full European Health Data Space (EHDS) implementation.

**Methods:**

The infrastructure was implemented using selected nationwide registries, including reimbursement, cancer, reproductive health, and mortality databases. Synthetic datasets were generated using registry-specific rule-based procedures designed to preserve database structures, variable formats, and selected longitudinal relationships while preventing reconstruction of real patient records. Researchers developed and tested SQL-based analytical workflows within the synthetic environment, whereas execution on primary NHIS databases and disclosure control review were performed exclusively by authorized personnel. The implementation was evaluated using predefined operational indicators, including user adoption, request volume, processing outcomes, transparency measures, and privacy-related incidents.

**Implementation (Results):**

The infrastructure enables the development and validation of SQL-based analytical workflows without direct access to primary patient-level data. Synthetic datasets representing approximately 0.04% to 0.26% of the selected NHIS registries were created while maintaining schema compatibility and selected analytical relationships. Between the platform’s public launch and July 23, 2026, a total of 170 users registered, 199 requests were submitted, and 184 requests have been processed (173 with data delivery). A total of 41 (20.6%) requests included consent to share SQL scripts, thereby supporting public transparency and reproducibility. Practical implementation was demonstrated through operational use cases in oncology, nephrology, cardiology, and vaccination research. No privacy-related incidents were reported during the evaluation period.

**Conclusions:**

The Czech NHIS synthetic data infrastructure demonstrates that privacy-preserving secondary use of national health data can be implemented through a General Data Protection Regulation (GDPR)–aligned governance framework that combines synthetic data, controlled execution on operational databases, disclosure-risk assessment, and transparent analytical workflows. Rather than serving as a statistically valid surrogate for inferential research, the infrastructure supports the reproducible development of workflows and the secure preparation of population-level analyses. As a fully operational national implementation aligned with the principles of the EHDS, the platform may provide a reference model for other countries developing privacy-preserving health data ecosystems.

## Introduction

### Background

In recent years, there has been a significant increase in demand for health data for reporting and analytical purposes, particularly from government bodies, research institutions, industrial partners, and commercial entities [1]. The main goal is to enable data-driven innovation in health care while protecting patient privacy, supported by initiatives operating within the legal framework of the relevant jurisdiction (eg, the General Data Protection Regulation [GDPR] in Europe or the Health Insurance Portability and Accountability Act [HIPAA] in the United States or the Health Information Act [HIA] in Canada) to ensure compliance with regulations. A promising solution to bridge this gap in data access is synthetic data, which are artificially generated data that simulate real patient information [2,3]. By generating realistic but not real patient records, national organizations can share and use health care datasets more freely without revealing sensitive personal data. Several national public platforms (eg, NHS England [4], France’s Health Data Hub [5], Finland’s Health Campus Turku [6], Germany’s BfArM Lab [7], Canada’s Children’s Hospital of Eastern Ontario Research Institute [8], and Statistics Netherlands [9]) integrated into health data infrastructures represent gateways to the world of real health data. These platforms explore synthetic data as a new feature used mainly for software testing, training AI models, sharing data, and expanding research datasets. Technologically, machine learning or statistical modeling is used mainly to generate synthetic data. The platforms differ in organizational models, data fidelity, and privacy levels, as well as in their maturity and adoption. Synthetic data replicate real-world health data structure and key characteristics without providing personal information. They enable robust analyses—such as algorithm development or patient pathway simulations—while protecting privacy. The European Health Data Space (EHDS) supports secure, cross-border data sharing and innovation. Proper cataloging ensures standard compliance, and such data can provide strategic insights into national health care sustainability, risks, and development needs [10].

### Synthetic Data Inside and Outside Medical Research

Synthetic data have emerged as an important approach for enabling data-driven innovation while protecting privacy, with applications across health research, AI, and health care analytics [11]. Studies have shown that synthetic datasets can approximate key properties of real-world medical data and support analytical development while reducing privacy risks [12]. Beyond structured health records, synthetic methods have also been applied to medical imaging and AI model development [13-16]. International experience further demonstrates their use in in silico clinical trials, epidemiological modeling, predictive analytics, and privacy-preserving collaboration between health care and research institutions [17-21].

Although these applications often focus on preserving statistical properties for analytical or inferential purposes, the National Health Information System (NHIS) implementation described in this report follows a different objective. The primary goal of the introduced system is to support the development, testing, and validation of analytical workflows. Consequently, the synthetic datasets were designed to preserve database structures and selected logical relationships rather than to serve as statistically representative surrogates of the original registries.

### Objectives

The objective of this manuscript is to describe the design, implementation, and operational deployment of the Czech national health data infrastructure, a novel access model for privacy-preserving use of national health data. It enables researchers to develop and validate analytical workflows in a synthetic environment and subsequently generate outputs from operational NHIS data under strict governance and disclosure control procedures. This approach creates an innovative pathway for accessing high-value health data while maintaining compliance with legal and privacy requirements. It demonstrates the continuing relevance of national privacy-preserving infrastructures during the transition toward the full implementation of EHDS.

## Methods

### Synthetic Data in the Czech Health System

The growing demand for the secondary use of health data must be balanced against privacy protection and regulatory requirements, including the GDPR and the emerging EHDS framework [22]. The NHIS Synthetic Data Infrastructure (SDI) was developed to address this challenge by enabling privacy-preserving access to high-value health data while minimizing the risk of reidentification [23]. The infrastructure is embedded within the Czech NHIS, operated by the Institute of Health Information and Statistics of the Czech Republic (UZIS), which provides comprehensive population-level coverage through the integration of 63 national health data sources (Figure 1), including health registries, reimbursement databases, health care provider registries, and supporting information systems [24,25]. Together, these components form a unified and interoperable platform for health system monitoring, research, and policymaking and provide the data backbone for a nationally implemented synthetic data access model.

**Figure 1.**
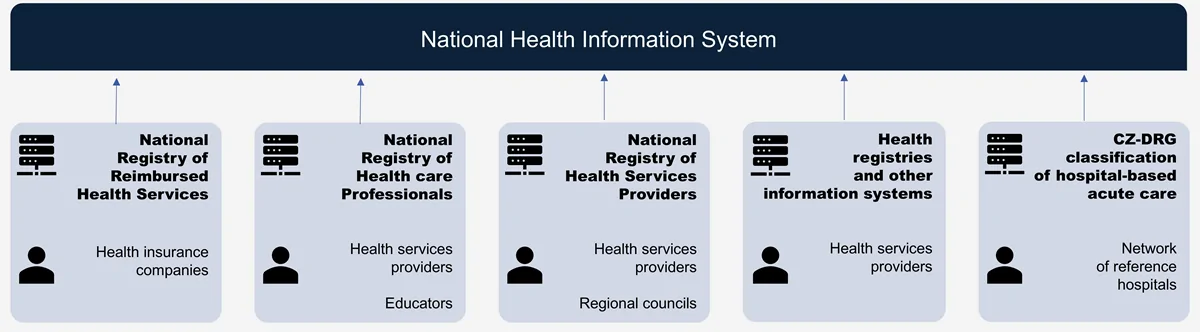
Core National Health Information System components and data sources [24]. CZ-DRG: Czech Diagnosis-related group.

Researchers develop and validate analytical workflows using computational scripts over synthetic datasets that reproduce registry structures and selected logical relationships while avoiding exposure of patient-level information. In contrast, execution on operational NHIS data is performed exclusively by authorized personnel under established governance and disclosure control procedures. The implementation is based on 3 core principles:

- Transparency is ensured by openly managing requests in the synthetic data system. A publicly available list contains each request with its subject, description, status, and applicant, ensuring accountability and oversight.
- Fairness is ensured by allowing each applicant to submit only one request at a time. They may submit another after it is processed (approved or rejected). This prevents monopolization and guarantees equal access for all.
- Privacy is maintained by designing the synthetic data environment to minimize disclosure risks by preventing direct access to primary NHIS databases and applying multilevel anonymization and GDPR-aligned disclosure control procedures to all released outputs.

### Creating Synthetic Databases

Synthetic datasets were generated separately for each NHIS registry using registry-specific, rule-based procedures developed by UZIS; no machine learning or generative AI models were used. The generation process (Textbox 1) preserved database schemas, table structures, data types, key relationships, and selected workflow-relevant logical rules while replacing all direct identifiers with randomly generated synthetic identifiers. Categorical variables were populated with permissible registry values, and continuous variables were populated with predefined registry-specific ranges, whereas original timestamps and operational identifiers were excluded. To support testing of cross-registry workflows, synthetic identifiers were consistently propagated across selected datasets, enabling controlled joins without linkage to real individuals or health care events. The resulting datasets were designed exclusively for the development and validation of SQL workflows, not as statistically representative surrogates for operational registries; all clinically or epidemiologically interpretable outputs are generated only after executing validated workflows on operational NHIS data.

Textbox 1. **Actions leading to synthetic data generation.**

- The attributes are divided into categorical and continuous attributes.
- Direct subject identifiers and the table’s primary key are excluded and replaced with their synthetic (randomly generated) variants.
- All unique values for categorical attributes are extracted from the primary table.
- The categories of each categorical attribute are often inserted into the synthetic table regardless of their actual distribution in the original data.
- The number of rows in a synthetic table usually corresponds to the largest number of categories among all categorical attributes.
- The absolute minimum and maximum values are selected from each continuous attribute and adjusted toward the mean (usually the 5th and 95th percentiles, respectively). This masks outliers that could lead to the identification of a specific data subject. The required number of values is randomly selected from the remaining original data.

### Structural Fidelity Assessment

Because the primary objective of the infrastructure is workflow development rather than inferential analysis, fidelity assessment focused on structural compatibility instead of statistical similarity. Validation included verification of schema equivalence, attribute compatibility, referential integrity, data type consistency, and successful execution of benchmark SQL workflows across both synthetic and operational NHIS environments.

The synthetic data warehouse contains artificially generated data covering selected recent time periods, representing approximately 0.1% of the volume of the primary production environment. For example, the National Registry of Reimbursed Health Services contains approximately 100,000 synthetic patients, 10 million health care receipts, and 20 million health care service records. To minimize disclosure risks while preserving analytical usability, the system includes only the most frequent health care entities and events derived from real NHIS statistics, including the 100 most frequent provider specializations; the 100 most frequent 3-character *International Classification of Diseases, Tenth Revision* (*ICD-10*) diagnosis groups; the 100 most frequent health care procedures; the 10 most frequent individually prepared medicinal products, dental devices, and transport services; 5 patient care categories; and randomly generated identifiers representing 1000 health care providers and 10,000 individual health care workplaces.

Statistical similarity between synthetic and operational datasets was not evaluated because preservation of underlying population distributions was not an intended design objective of the infrastructure. Instead, the successful execution of analytical workflows in both environments served as the principal validation criterion.

### Disclosure-Risk Controls and Governance

The synthetic environment contains only artificially generated records and therefore does not require disclosure assessment of analytical results produced solely within the synthetic database. However, outputs generated by executing validated workflows on operational NHIS data are subject to mandatory disclosure control review. The review process includes technical, methodological, legal, and privacy assessment by authorized UZIS personnel and the Data Protection Officer. Reidentification risk is evaluated through minimum cell size controls, proportionality assessment, and verification of compliance with approved purposes of use [26-31]. As a standard rule, outputs containing subgroups with fewer than 10 observations are suppressed, aggregated, or otherwise modified before release. Final publication also requires approval from UZIS management.

### Access Workflow

Researchers obtain access to the synthetic NHIS environment through a one-time registration process, during which they develop, test, and validate SQL-based analytical workflows without access to operational patient-level records. Completed SQL scripts, including metadata descriptions, are submitted through a formal request procedure and executed exclusively by authorized UZIS personnel on operational NHIS databases. Figure 2 shows the interaction between the user requesting data and the data manager representing UZIS. The resulting outputs undergo disclosure control review and may subsequently be released as controlled, deidentified, patient-level datasets or aggregated analytical outputs. Metadata describing submitted requests and their status are published through a public request overview to support transparency and reproducibility.

Although UZIS provides the infrastructure, governance framework, and privacy protection mechanisms required to minimize disclosure and reidentification risks, responsibility for the analytical design, interpretation, scientific validity, and subsequent use of the released outputs remains solely with the applicant. The resulting analytical dataset or output is therefore considered the applicant’s intellectual work. At the same time, UZIS does not guarantee the analytical validity, completeness, statistical representativeness, or appropriateness of conclusions derived from either the synthetic environment or the released results.

**Figure 2.**
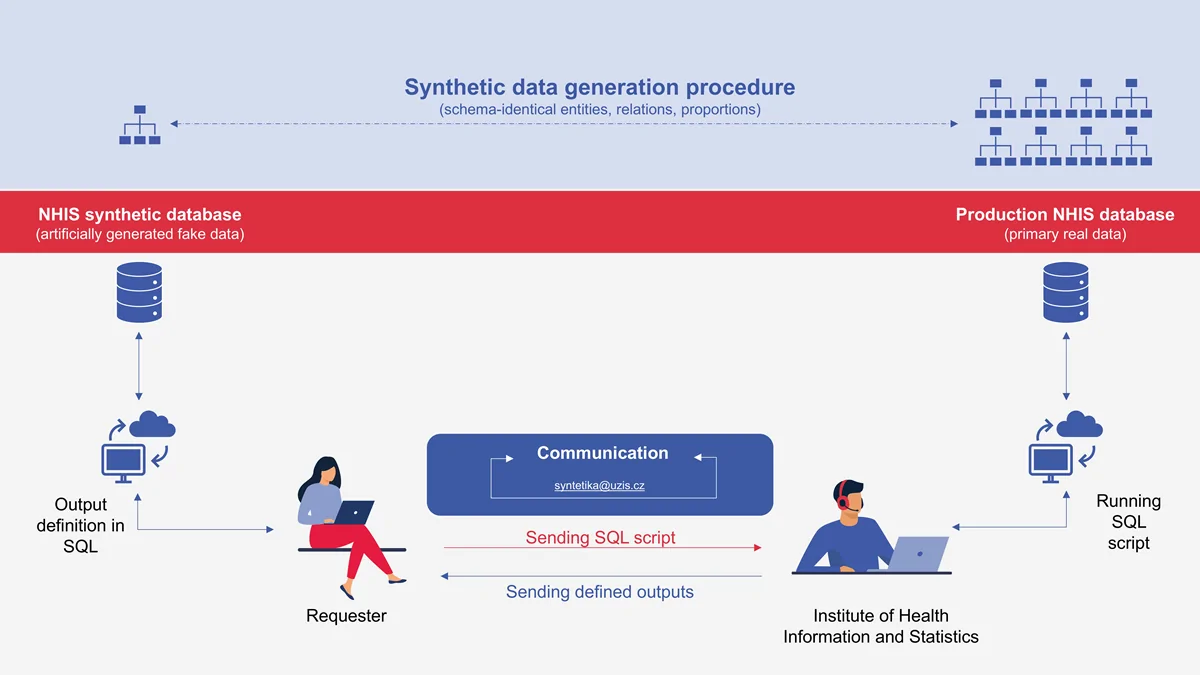
National Health Information System (NHIS) synthetic data: communication scheme.

### Evaluation Framework

The implementation was evaluated using routinely collected operational monitoring data covering the period from the public launch of the infrastructure in May 2025 through July 2026. Consistent with the project’s implementation-oriented objective and the workflow described above, the evaluation focused on operational performance, adoption, governance effectiveness, transparency, and privacy protection rather than on the content, scientific conclusions, or analytical validity of user-generated outputs. Researchers independently develop computational scripts within the synthetic environment, whereas UZIS is responsible for the technical control, execution on operational databases, and disclosure-risk assessment before release. Accordingly, UZIS ensures compliance with governance and privacy requirements, but does not validate, interpret, or guarantee the scientific quality, completeness, or appropriateness of datasets and analytical outputs generated from submitted SQL workflows.

Predefined evaluation outcomes therefore included the number of registered users, the number of submitted requests, processing outcomes, processing times, the proportion of publicly shared SQL workflows, and the number of privacy-related incidents. These indicators were selected to assess practical utility, sustainability, governance effectiveness, transparency, and acceptance of the infrastructure as a national mechanism for privacy-preserving secondary use of health data.

### Ethical Considerations

This study describes the implementation and operational evaluation of a national SDI within the Czech NHIS and did not involve direct interaction with human participants. Researchers were not provided with access to identifiable patient-level data; analytical workflows were developed using synthetic datasets and executed on operational NHIS databases exclusively by authorized UZIS personnel under established governance procedures. The entire infrastructure therefore did not constitute human subjects research requiring informed consent or independent ethics committee review under the applicable Czech legal framework governing the NHIS and the secondary use of health data for public health, statistical, and research purposes. All requests were subject to methodological, legal, and disclosure control review. No identifiable patient information is reported in this manuscript, and no privacy breaches or unauthorized disclosures were identified during the evaluation period.

## Implementation (Results)

### General Overview

The infrastructure currently provides synthetic representations of selected NHIS registries covering reimbursement, reproductive health, cancer, and mortality data (Table 1). Synthetic datasets represent 0.04% to 0.26% of the corresponding operational registries while preserving database structures and workflow-relevant relationships required for SQL-based analytical development.

The infrastructure demonstrated sustained operational use through pilot testing and routine use by the health data community (Table 2). Between May 15, 2025, and July 23, 2026, a total of 170 users registered (n=119, 69.8% from the commercial sector; n=44, 25.6% individual users; and n=9, 5% from civic initiatives and research organizations), 49 users submitted at least one request, and 199 requests were received, of which 184 reached an outcome (Table 3).

**Table 1.** Sizes and proportions of selected National Health Information System (NHIS) registries and their synthetic derivatives.

	Coverage period used for the synthetic dataset design	Primary NHIS data coverage period	Synthetic data, n (%)	Primary NHIS data, n (%)
National Registry of Reimbursed Health Services	2015‐2019	2010‐2024	14,201,812 (0.09)	16,200,834,967 (100)
National Registry of Reproductive Health	2015‐2019	1994‐2023	1163 (0.04)	3,054,931 (100)
National Cancer Registry	1977‐2022	1977‐2023	6714 (0.21)	3,138,236 (100)
Death Records Database	2015–03/2020	1987‐2023	10,777 (0.26)	4,193,272 (100)

**Table 2.** Overview of the synthetic data system.

Characteristic	Value
Registered users, n	170
Submitted requests^a^, n	199
Users with at least one request, n	49
Number of requests per registered user who submitted at least one request, mean (SD)	4.06 (7.50)
Number of business days required to process the application, mean (SD)	15.63 (10.37)
Number of privacy-related incidents, n	0

a Of the 199 submitted requests, 184 had been processed and 15 were in progress. Processed requests included requests with final outcomes (delivered, canceled, rejected, or returned for revision), whereas requests marked “in progress” were excluded.

**Table 3.** Processing status of submitted requests in the synthetic data system (N=199).

Request status	Requests, n (%)
Closed with data delivery	173 (86.9)
Canceled	7 (3.5)
Rejected	4 (2.0)
In progress	15 (7.5)

Public sharing of SQL scripts was enabled for 41 (20.6%) requests via the NZIS Open Journal [32], thereby supporting transparency and reproducibility. No privacy-related incidents, disclosure control failures, or confirmed reidentification events were reported.

Collectively, these implementation outcomes demonstrate sustained adoption, operational feasibility, and effective governance of the infrastructure. The combination of continued user engagement, successful request processing, public sharing of SQL workflows, and the absence of privacy-related incidents supports the practical viability of the NHIS SDI as a national mechanism for privacy-preserving secondary use of health data.

### Use Cases

The following use cases illustrate practical deployment of the infrastructure in real-world analytical projects. It is being developed with the aim of facilitating the secondary use of health data (particularly from the perspective of patients) in accordance with the EHDS framework, its rules, and objectives.

#### Use Case 1: Patient Pathway for the Treatment of Cancers

Longitudinal health care data from national reimbursement records can be used to reconstruct patient pathways while preserving privacy. In this use case, a workflow was developed to analyze the early care pathway of patients with newly diagnosed lung cancer using administrative claims data and predefined procedural and diagnostic codes. The synthetic environment enabled development, testing, and technical validation of the workflow without access to operational patient-level data. Following successful technical validation, the workflow was executed on NHIS databases and generated clinically interpretable outputs in accordance with established disclosure control procedures. This use case demonstrates that the SDI can support the development of reproducible patient pathway analyses using national health data while maintaining privacy protection and governance requirements.

#### Use Case 2: Dialysis Patient Pathway Through the Health Care System

In collaboration with a clinical expert society, an analytical workflow was developed to characterize health care pathways of patients with chronic kidney disease and dialysis treatment. The workflow identified patients undergoing selected dialysis-related procedures and linked information on health care use, specialist consultations, pharmacotherapy, kidney transplantation, and mortality. Development and validation of the SQL script were performed within the synthetic environment before execution on the primary NHIS databases. Following disclosure control review, the resulting dataset was released to the applicant and contained longitudinal information required for descriptive analyses of treatment pathways and outcomes (Table 4).

**Table 4.** Structure of the resulting dataset.

Attribute of the given dataset	Example value
Anonymous patient ID	1
Month and year of treatment	202001
Aggregated procedure codes by patient ID and date	81499, 11022, 81365, 81329, 18550
Aggregated prescribed pharmacotherapy codes by patient ID and date	V06XX, H05BX02
Prescribing medical specialty in chronological order	128
Month and year of death	202001
Primary cause of death according to the *ICD-10^a^*	A418

a *ICD-10*: *International Classification of Diseases, Tenth Revision.*

#### Use Case 3: Burden of Cardiovascular Diseases

A further use case involved developing analytical workflows to support the National Cardiology Information System, which integrates data from multiple NHIS sources to monitor cardiovascular disease burden and health care delivery. The workflows were developed and validated in a synthetic environment and subsequently executed on the primary NHIS databases. One of the resulting outputs provided population-level estimates of cardiovascular diseases, stratified by demographic and geographic characteristics. In contrast, another summarized the number of treated patients across major cardiovascular diagnostic groups between 2015 and 2022 (Table 5). This use case demonstrates the infrastructure’s ability to support the preparation of nationwide analytical outputs from multiple linked health registries [32-35].

**Table 5.** Total number of patients treated for cardiovascular diseases in the Czech Republic (source: National Registry of Reimbursed Health Services).

Disease (*ICD-10^a^* code)	2015	2016	2017	2018	2019	2020	2021	2022
Diseases of the circulatory system (I00-I99), n	2848	2890	2898	2900	2931	2959	2919	2895
Diseases of the circulatory system (I00-I99), excluding vascular diseases of the brain (I60-I69), n	2793	2835	2842	2845	2877	2910	2867	2844
Acute coronary syndrome (I20.0 and I21-I22), n	48,724	47,034	45,946	44,482	43,494	42,549	42,539	39,659
Ischemic heart disease (I20-I25), n	612,104	596,061	577,397	556,696	541,006	525,400	497,363	469,997
Heart failure (I50, I11.0, I13.0, I13.2, I25.5, I42.0, I42.9, and R57.0), n	115,625	117,883	121,131	121,084	125,547	127,478	130,878	130,841
Valvular disease (I05-I08 and I33-I39), n	98,405	101,846	104,910	106,745	109,862	103,230	107,483	108,410
Cardiomyopathy (I42), n	19,476	19,896	20,063	20,233	20,646	19,937	20,429	20,078
Conduction disorders and arrhythmias (I44, I45, and I47-I49), n	367,458	380,802	394,699	405,743	416,420	406,463	419,581	421,748

a *ICD-10*: *International Classification of Diseases, Tenth Revision.*

#### Use Case 4: Meningococcal B Vaccination

This use case evaluated the feasibility of using the SDI to analyze vaccination uptake and outcomes associated with meningococcal B vaccination using linked national health data. The analytical workflow was developed and validated in a synthetic environment before being executed on operational NHIS databases under established governance procedures. Following disclosure control review, the resulting outputs provided evidence to support the assessment of vaccination coverage and related health care outcomes. This use case demonstrates that the SDI can support the development of reproducible population-based analyses relevant to preventive health care and public health decision-making while maintaining privacy protection requirements.

## Discussion

The results demonstrate the successful implementation of a national SDI supporting privacy-preserving analytical workflows. Sustained operational use, successful processing of analytical requests, and the absence of privacy-related incidents indicate operational validity. At the same time, the infrastructure provides a reproducible environment for workflow development and secure execution on operational NHIS databases.

### Operational and Privacy Challenges

Despite its successful adoption, the infrastructure faces several challenges. First, request processing requires technical, methodological, legal, and disclosure control review of each submitted workflow, creating capacity constraints as demand grows. Similar scalability limitations have been reported in other data sharing and federated research infrastructures [35]. Second, increasing transparency through publication of outputs and SQL workflows may also increase cumulative disclosure risks when multiple outputs are combined. Previous studies have shown that linking datasets may enable reidentification or inference of sensitive attributes despite anonymization safeguards [36]. Consequently, all outputs generated from operational NHIS data undergo a multistep disclosure-risk assessment before release [37]. Ongoing methodological development therefore focuses on semiautomated privacy-risk assessment and future AI-assisted disclosure control procedures. Third, rapid advances in AI may alter the balance between data utility and privacy protection. Synthetic data and privacy-preserving techniques that appear sufficient today may become vulnerable to future inference or reconstruction attacks. Evidence that synthetic data–based models can achieve performance comparable to models trained on real-world data [38,39] highlights the need for continuous reassessment of governance frameworks, anonymization methods, and disclosure control procedures. Within the NHIS infrastructure, privacy protection is therefore treated as an ongoing governance process rather than a one-time technical solution.

### User Responsibility and Transferability

The NHIS synthetic environment is based on a decentralized model in which users independently design computational workflows and analyze released outputs for descriptive and analytical purposes. Consequently, the quality and validity of analytical outputs depend on individual users’ expertise, highlighting the importance of data literacy, methodological guidance, and reproducible workflows [40]. The Czech NHIS infrastructure demonstrates a practical, GDPR-compliant model for the secondary use of health data, aligned with emerging EHDS principles [41]. By combining synthetic datasets, controlled execution on operational databases, disclosure control review, and transparent SQL-based workflows, the platform provides a transferable framework that may inform similar implementations in other countries. Wider adoption will, however, require harmonized approaches to governance, interoperability, and disclosure-risk assessment across Europe.

### Conclusions

The Czech NHIS SDI demonstrates that privacy-preserving secondary use of national health data can be implemented through a governance framework that combines synthetic data, controlled execution on operational databases, disclosure-risk assessment, and transparent analytical workflows. The platform enables development, testing, and validation of SQL-based analytical workflows without direct access to patient-level data.

The infrastructure was designed to support workflow development rather than statistical replication of source registries. Consequently, clinically interpretable outputs are derived exclusively from primary NHIS databases following execution of validated workflows and disclosure control review.

Evaluation of the platform demonstrated successful implementation, as evidenced by 170 registered users (data scientists and analysts), reflected in sustained user adoption, processing of analytical requests, public sharing of SQL workflows, and the absence of privacy-related incidents. As a fully operational national implementation aligned with GDPR and EHDS principles, the infrastructure may serve as a transferable reference model for privacy-preserving health data ecosystems.

## Supplementary material

10.2196/80683 Checklist 1 iCHECK-DH checklist

## Abbreviations

GDPR: General Data Protection Regulation
HIA: Health Information Act
HIPAA: Health Insurance Portability and Accountability Act
*ICD-10*: *International Classification of Diseases, Tenth Revision*
NHIS: National Health Information System
SDI: synthetic data infrastructure
UZIS: Institute of Health Information and Statistics of the Czech Republic
