# Supplementary material for: Implementation of a National Synthetic Data Infrastructure for Privacy-Preserving Analytical Workflows in the Czech National Health Information System: Development and Evaluation Study
Source: JMIR Med Inform. 2026 Nov 10;14:e80683. doi: 10.2196/80683 (PMC13756756; doi:10.2196/80683)
Supplement: Supplementary file 1 [file medinform-v14-e80683-s001.docx]

Implementation of a National Synthetic Data Infrastructure for Privacy-Preserving Analytical Workflows in the Czech National Health Information System: Development and Evaluation Study

iCHECK-DH checklist

| **1. Implementation context** | The implementation was conducted within the Czech National Health Information System (NHIS), a nationwide health data infrastructure operated by the Institute of Health Information and Statistics of the Czech Republic (UZIS). The NHIS integrates 63 national health data sources, including health registries, reimbursement databases, health care provider registries, and supporting information systems. |
| --- | --- |
| **2. Health need/problem addressed** | The implementation addresses increasing demand for secondary use of national health data while maintaining compliance with privacy, data protection, and disclosure-control requirements. Direct access to operational patient-level NHIS databases is restricted. |
| **3. Digital health intervention** | The intervention is a national synthetic data infrastructure that enables researchers to develop, test, and validate SQL-based analytical workflows using synthetic representations of selected NHIS registries without direct access to operational patient-level data. |
| **4. Objectives** | The objective was to design, implement, deploy, and operationally evaluate a national privacy-preserving infrastructure that supports the development of reproducible analytical workflows and the secondary use of health data. The implementation also examines its relevance during transition toward the European Health Data Space (EHDS). |
| **5. Target users** | Intended users include researchers, data scientists, analysts, governmental institutions, research organisations, industry partners, and other users who require secondary analysis of national health data and multidisciplinary expert domain knowledge. Operational data showed 170 registered users, including commercial, individual, civic, and research users. |
| **6. Technology and architecture** | Synthetic datasets reproduce registry schemas, table structures, data types, selected key relationships, and workflow-relevant logical rules. Users develop SQL workflows in the synthetic environment; validated scripts are subsequently executed on operational NHIS databases exclusively by authorised UZIS personnel. |
| **7. Data and interoperability** | Synthetic representations currently cover reimbursement, reproductive health, cancer, and mortality data. Synthetic identifiers enable controlled cross-registry joins, while schema and attribute compatibility allow workflows developed in the synthetic environment to be transferred to operational NHIS databases. |
| **8. Privacy, security and governance** | Governance is based on transparency, fairness, and privacy. Users have no direct access to primary NHIS databases. Outputs from operational data undergo technical, methodological, legal, privacy, and disclosure-control review. Small cells with fewer than 10 records are suppressed, aggregated, or modified before release. |
| **9. Regulatory environment** | Implementation operates within the Czech legal framework governing NHIS and is aligned with GDPR requirements and emerging EHDS principles. |
| **10. Implementation process** | Users register once, develop and validate SQL scripts using synthetic data, submit completed workflows and metadata, and UZIS personnel execute approved workflows on operational databases. Outputs subsequently undergo disclosure-control review before release. |
| **11. Stakeholders and responsibilities** | UZIS provides and governs the infrastructure and performs technical execution, legal review, and disclosure-risk assessment. Users remain responsible for analytical design, interpretation, scientific validity, and subsequent use of released outputs. Clinical and industry stakeholders contributed to selected implementation use cases. |
| **12. User access and workflow** | Access is based on registration and a formal request procedure. Users work with synthetic data rather than operational patient-level records and submit validated SQL scripts for controlled execution by authorised personnel. Request metadata and status information are made publicly available. |
| **13. Training/user support** | The manuscript describes the user workflow and methodological guidance but does not provide a sufficiently explicit description of onboarding, training materials, helpdesk support, or user documentation. |
| **14. Implementation period and maturity** | Operational evaluation covered the period from the public launch in May 2025 through July 2026. The infrastructure is described as a fully operational national implementation rather than a pilot-only system. |
| **15. Evaluation approach** | Evaluation used routinely collected operational monitoring data and predefined indicators, focusing on adoption, operational performance, governance effectiveness, transparency, and privacy protection, rather than on the scientific validity of user-generated analyses. |
| **16. Implementation outcomes** | The implementation achieved 170 registered users, 199 submitted requests, 184 requests reaching an outcome, 173 requests closed with data delivery, and 41 requests with public SQL sharing. No privacy-related incidents were reported during the evaluation period. |
| **17. Demonstrated use / real-world application** | Four implementation use cases are reported: lung cancer patient pathways, dialysis care pathways, cardiovascular disease burden, and meningococcal B vaccination. These demonstrate workflow development in synthetic data followed by controlled execution on operational NHIS data. |
| **18. Challenges and lessons learned** | Identified challenges include increasing capacity requirements for technical, methodological, legal, and disclosure review; cumulative disclosure risk from multiple outputs; and evolving privacy risks associated with advances in AI. Privacy protection is consequently treated as an ongoing governance process rather than a one-time technical measure. |
| **19. Sustainability, scalability and transferability** | The infrastructure demonstrates sustained operational use, but increasing demand creates scalability challenges. The combination of synthetic data, controlled execution, disclosure review, and transparent workflows is proposed as a potentially transferable model for other national health data ecosystems. However, wider implementation requires harmonised governance, interoperability, and disclosure-risk approaches. |
| **20. Ethical considerations/conflicts / funding** | The study did not provide researchers with identifiable patient-level information and involved no direct interaction with human participants. The manuscript reports the applicable ethical rationale, governance safeguards, funding, author contributions, conflicts of interest, and data availability. |
